# Do Fans Impact Sports Outcomes? A COVID-19 Natural Experiment^∗^

**DOI:** 10.1177/15270025221100204

**Published:** 2023-01

**Authors:** Jeffrey Cross, Richard Uhrig

**Affiliations:** 1 2576 Hamilton College, Utica, NY, USA; 2 8786 University of California, Santa Barbara, CA, USA

**Keywords:** home field advantage, soccer, COVID-19, fan attendance, Z2

## Abstract

This paper studies the effect of fan attendance on home field advantage in top European soccer leagues. We exploit exogenous variation in the level of fan attendance driven by COVID-19 mitigation policies and find that the home field advantage, as measured by home minus away (expected) goals, is reduced by more than 50% across the English Premier League, German Bundesliga, Italian Serie A, and Spanish La Liga. This leads to a decrease in probability for a home win, indicating that these goals are pivotal with respect to match outcomes.

## Introduction

Home teams generally outperform away teams in a wide variety of sports. This stylized fact is well-established and known as home field advantage. Over the course of the 2008–2009 through 2018–2019 season in four top European leagues, home teams outperformed away teams in goal difference by 0.38 goals and win percentage by 17 percentage points.^1^ This advantage is driven by various factors that are typically broken down into three main channels: travel fatigue, venue familiarity,^2^ and crowd support. It is an empirical challenge to separately identify each effect. While fan attendance is indeed measurable, there are endogeneity concerns regarding the impact of crowd attendance on team performance; better teams typically have bigger stadia and higher attendances, raising concerns about reverse causality.

In this paper, we exploit exogenous variation, driven by the COVID-19 pandemic, in the level of fan attendance. We leverage the fact that four of the top European soccer leagues implemented no-fans policies for the last quarter or so of the 2019–2020 season. These policies cause an exogenous downward shock in attendance levels at soccer games. We find that these no-fans policies decrease the home field advantage in European soccer as measured by goal difference, expected goal difference, and the probability of a home win. These results are robust to inclusion of various fixed effects as well as controls for weather, team strength, and local exposure to COVID-19.

We explore both the causes and implications of this change in home field advantage. Using the expected goals metric, we show that the decrease in home field advantage is driven by home teams playing worse relative to away teams after the implementation of the no-fans policy, as opposed to scoring relatively fewer goals on the same quality and quantity of goal-scoring opportunities. Our results show that the change in home field advantage decreases the probability a home team wins the game by 5.4 percentage points. We find that there is no effect on the probability of a draw between the home team and the away team as decreases in wins led to a direct increase in losses for the home team. This finding suggests that fans have a symmetrically pivotal impact on the outcome of games — the same number of games are shifted from home win to draw as are shifted from draw to home loss.

Of all the potential sports leagues with which to examine the impact of fan attendance on home field advantage, the European soccer leagues discussed here provide the best natural experiment. The rules of the game were not materially affected in any way that might plausibly impact home field advantage.^3^ While games were delayed between two and three months, no games were canceled or relocated. This is in stark contrast to both other soccer leagues (Major League Soccer in the United States, European club tournaments such as the UEFA Champions League and the UEFA Europa League), international soccer matches, and North American leagues in other sports (National Basketball Association, Major League Baseball, National Football League, etc.).^4^

This paper contributes to a rich literature focused on identifying the effects of crowd support, travel fatigue, and venue familiarity on the home field advantage. Broadly speaking, the literature on crowd support prior to COVID-19 can be split into two strands. The first strand uses variation in turnout by country and league and generally finds larger advantages for the home team associated with higher fan attendance (Agnew & Carron, 1994; Clarke & Norman, 1995; Pollard & Pollard, 2005). In the second strand, two recent papers leverage quasi-experimental variation in fan attendance to overcome endogeneity concerns. Belchior (2020) uses randomized game times in Brazilian football and finds that fan attendance levels have no effect on home field advantage. Ponzo and Scoppa (2018), on the other hand, provide evidence for the importance of crowd support using same-stadium derbies.^5^ In this setting, both teams are familiar with the stadium and do not suffer travel fatigue, but tickets are allocated differently to the home and away teams. Using 20 years of Serie A data and 5 same-stadium derbies, they find that the home team is 15 percentage points more likely to win than the away team, significantly lower than the 25 percentage point advantage in normal matches. We generalize the results of Ponzo and Scoppa (2018) and also find that fans drive a large component of home field advantage in soccer beyond same-stadium derbies in Italy.

Various papers exploit the variation in attendance from COVID-19 to identify the effect of fans on home field advantage. The vast majority of these papers find that the no-fans policies decreased home field advantage (Ferraresi & Gucciardi, 2020; Fischer & Haucap, 2021; Scoppa, 2021), but some find statistically insignificant effects (Bryson et al., 2021; Reade et al., 2021).^6^ Some differences in results can be attributed to heterogeneous effects by country (Benz & Lopez, 2021) and by division (Fischer & Haucap, 2021). We focus on the top divisions in England, Germany, Italy, and Spain, which are the top-ranked leagues in European football, and include a rich set of controls to best isolate the effect of fans on home field advantage in the most important leagues.

This paper differs from the recent literature on home field advantage during COVID-19 in a few important dimensions. First, we examine the effect of the no-fans policy not just on realized goals but also on expected goals. Expected goals are a measure of team performance that accounts for the quantity and quality of shots, which allows us to better isolate the impact of fans on performance. Second, we include controls for various measures of COVID-19 prevalence. Although it is difficult to know ex-ante how COVID-19 would affect home field advantage directly, we control for potential heterogeneous regional effects using data on COVID-19 cases from Carleton et al. (2021). Third, we control for differences in weather relative to when these games would have otherwise been played; the hiatus in the 2019–2020 season meant that games originally scheduled for March through May were instead played in May through August. Therefore, the temperature during these games was hotter than would have otherwise been expected, and this may lead to differences in home field advantage. By controlling for geospatial variation in cases and intertemporal changes in temperature, we are able to better account for the direct effect of the pandemic and the delay in the season.

Our results indicate that between 35% and 45% of the home field advantage can be attributed to non-fan factors of travel fatigue and venue familiarity. Oberhofer et al. (2010) analyze the effect of distance on home field advantage in the Bundesliga and find that the performance of the away team decreases with distance traveled. Similar effects have been found in the English Premier League (Clarke & Norman, 1995), U.S. National Football League (Nichols, 2014), and Australian Football League (Goumas, 2014), some of which also implicate crossing time zones as one channel through which travel may affect home field advantage. Familiarity also likely plays a role in the residual home field advantage as players on the home team are more comfortable in their own stadium. This has an especially large effect when the field is unusually large or small or has an artificial surface (Barnett & Hilditch, 1993; Clarke & Norman, 1995; Dowie, 1982). To causally identify the effect of familiarity, researchers have used team stadium moves that reduce familiarity for the home team. Evidence on this effect is mixed; Pollard (2002) finds a drop in home field advantage after moving, while Loughead et al. (2003) finds no effect. In this paper, we are not able to separately disentangle these two non-fan factors, but our work does imply that crowd support is the largest driver of home field advantage.

This paper is organized as follows: Section 2 provides background information and describes the data used. Section 3 discusses threats to identification. Section 4 explains the empirical strategy. Section 5 highlights the key results of our analysis. Section 6 concludes.

## Background and Data

In this section, we describe the five major European soccer leagues, their response to COVID-19, and the data utilized in the empirical analysis.

### European Soccer and COVID-19

The top five ranked professional football leagues in the Union of European Football Associations (UEFA) are the Spanish La Liga, English Premier League, Italian Serie A, German Bundesliga and French Ligue 1. These leagues have dominated both European soccer and global sports revenues in recent history.^7^ In 2017–2018, these five leagues generated approximately 17.4 billion dollars (USD) in revenue, which is more than the total GDP of Jamaica and double the total revenue of the NFL, highlighting the importance of these leagues relative to other sports and the global economy.

Seasons for all of the major European soccer leagues begin in August and end the following May. In the 2019–2020 season, however, the normal schedules were interrupted when COVID-19 was declared a pandemic by the World Health Organization on March 11, 2020. Amid concerns for player and staff safety, all major European leagues halted play from that date until mid-May at the earliest, with some slight variation by country.^8^

Four of the five leagues resumed play with a restrictive set of protocols.^9^ Many of the policies instituted across the leagues were similar during the season,^10^ but with some variation in the lead- up.^11^ In addition to testing and quarantine policies, each league allowed for two extra substitutions per match and instituted water breaks in order to alleviate the burdens of the hiatus and warmer weather. No games were canceled or moved, but all four of the leagues decided to play without any fans in attendance.

A brief summary of the timeline, on-field policy changes, and primary method of travel for each league is shown in Table 1. All of the leagues stopped at about the same time, but play resumed at different points of time for each league. The Bundesliga resumed play slightly over 2 months after the initial pause, while the other leagues waited approximately 3 months before resuming play. The mitigation policies were successful, no matches were canceled or rescheduled, and very few players missed games.^12^

**Table 1.** Covid Summary by League.

	Bundesliga	La Liga	Premier league	Serie A
Last Game before Pause	March 11	March 10	March 9	March 9
First Game after Pause	May 17	June 11	June 17	June 20
Days between Games	66	93	100	103
Games Canceled	0	0	0	0
On-Field Changes	*<*—————– 5 substitutes, Water Breaks —————–*>*			
Away Travel	Multiple buses	Travel organized in-house	Fly, bus, or personal vehicles	Multiple buses
Fans Allowed?	No	No	No	No

Notes: This table includes some basic information on how COVID-19 impacted each of the leagues. All of the leagues stopped at about the same time, but restarts were staggered. No games were canceled or postponed after the restart in any of the four major leagues.

### Schedule Construction in European Soccer

Teams in the five soccer leagues discussed in this paper all play what is typically called a “balanced” schedule. Every team plays each other team in the same league exactly twice, once at home and once away. Therefore, each team plays 2 *×* (*n* *−* 1) games in a season, where *n* is the number of teams in that league.^13^ Games against the same opponent are spaced throughout the season. In each of these leagues, a certain team will play each potential opponent once in the first half of the season and once in the second half. These two games are often called “reverse fixtures” since they contain the exact same teams but are played at opposite locations and times in the season. In some leagues, such as the Bundesliga, the order of opponents is exactly the same in each half of the season.^14^ For example, if Bayern Munich plays at Borussia Dortmund in each team's first game of the season, then Borussia Dortmund will play at Bayern Munich in the eighteenth game of the season.

Games are randomly allocated subject to some restrictions. These restrictions are in place to prevent long strings of home or away games for any given team and to avoid concurrent home games between two teams in the same metropolitan area. If games were perfectly randomly allocated, then there would occasionally be long strings of home or away games for a given team. If this were the case, it could present problems for analysis; results could be driven by randomness in the schedule rather than any changes in home field advantage. In the last ten matches of each league discussed here, every single team played between four and six home games, which miti- gates the concern that the home field advantage in the back part of the season is driven by a few teams with many home games.

### Expected Goals

Due to randomness, human error, and occasional moments of athletic brilliance, the realized score of a match is a noisy signal for which team actually played better over the course of 90 minutes. In order to mitigate this noise, we focus on expected goals, or xG, which measure the quantity and quality of each team's chances to score; they have been shown to better predict future performance and more closely track team actual performance than realized goals (Rathke, 2017). Expected goals are calculated by summing the ex ante probabilities that each shot, based on its specific characteristics and historical data, is converted into a goal.^15^ For example, if a team has four shots in a game, each with a scoring probability of 0.25, then their expected goals for the match would sum to 1. However, their realized goals could take any integer value from 0 to 4.^16^

Note the important difference between expected goals and realized goals in their respective data generating processes. Realized goals effectively transform the expected goals probabilities, in which *p* can take on any value between zero and one, into a Bernoulli variable with probability of success *p* equal to the expected goals metric. This process of “converting” chances into goals is subject to increased randomness, and we therefore focus on expected goals as the more precise measure of team performance.

To determine if fans impact the quantity and quality of chances created, we abstract from realized goals and instead use expected goals for each team as our primary measure of performance. We restrict the panel to years for which the expected goals data are available and estimate the effect of the no-fans policy on home field advantage as measured by expected goal difference. Importantly, this allows us to identify the effect of fans on quality of play, as measured through shot quality and quantity, rather than the noisier signal of realized goals.

### Data Description

We use match data from the soccer statistics website FBref,^17^ which provides information on all matches in the five major European soccer leagues going back at least 10 years. Our panel starts with the 2009–2010 season and thus includes 10 years of data prior to the 2019–2020 season and 15,906 matches overall. The data are slightly more limited for expected goals, which are only available on FBref starting in the 2017–2018 season.

We control for daily weather conditions at the location of the match using European Climate Assessment and Dataset (ECA&D) (Klein Tank et al. 2002).^18^ Game temperatures are then assigned weather data by matching the closest station-level daily average temperatures.^19^

**Figure A1.**
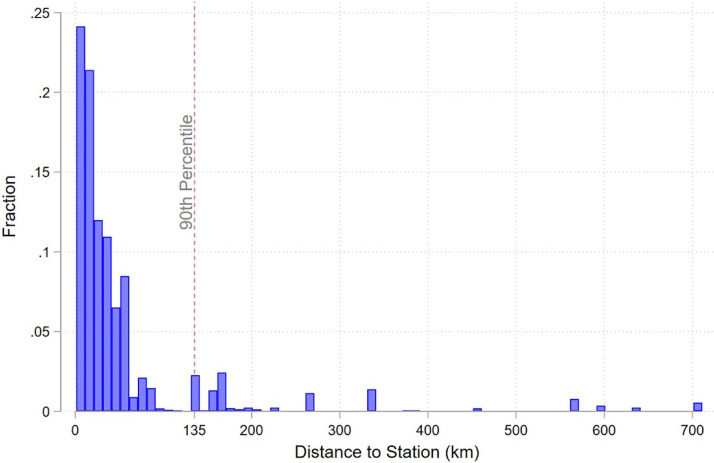
Distance from Stadium to Station. Note: This figure shows the distribution of distances between the stadium and stations. The gray line indicates the 90th percentile, which is 135 kilometers.

To measure the direct effect of COVID-19 across space, we use one of the most spatially- resolved global dataset of daily confirmed COVID-19 cases from Carleton et al. (2021).^20^ Combining the geographic location of each stadium with the geographic regions in the COVID-19 dataset, we calculate three different measures for the effect of COVID-19 on an area as of March 31, 2020: cumulative cases, new cases, and total cases per capita. For each of these measures, we calculate the difference between the home and away team and will use this as a measure of differences in the effect of the virus in the area of the home team compared to the away team.

### Summary Statistics

Table 2 presents key summary statistics aggregated across all four of the top five European soccer leagues that resumed play in May and June of 2020. Almost all match data is from the 2009–2010 season through the 2019–2020 season. The lone exception is the expected goals metric, which is only available starting in the 2017–2018 season. As a measure of distance traveled, we calculate the linear distance between the home and away team stadiums.

**Table 2.** Summary Statistics for All Leagues.

	Mean	Standard Error	Minimum	Maximum
Attendance (thousands)	32.52	18.74	0.01	259.30
Stadium Distance (km)	339.05	260.14	0.00	2259.93
Home Team Goals	1.57	1.32	0.00	10.00
Home Team Expected Goals	1.48	0.79	0.00	5.80
Away Team Goals	1.19	1.17	0.00	9.00
Away Team Expected Goals	1.19	0.71	0.00	5.30
Home-Away Goals Difference	0.38	1.83	−9.00	8.00
Home-Away Expected Goals Difference	0.29	1.15	−5.10	5.50

Notes: This table shows the summary statistics for all of the leagues. An observation is a game and stadium distance for each game is the distance between the two home stadia. Expected goals are only calculated for the 2017–2018 season onwards, but all other variables start in the 2009–2010 season.

There are two things worth highlighting from Table 2. First, home teams generally score more than away teams, which is reflected in a home minus away goal difference of 0.38. Second, there is a similar advantage to home teams in the home minus away expected goals difference. This highlights that the difference in actual goals reflects differences in goal-scoring opportunities. In other words, this table shows the core of what the home field advantage is — a difference in play between home and away teams, which then manifests in goals and match outcomes.

The average home field advantage, attendance, and distance between teams is different across each of the four leagues. In Table 3, we break down the summary statistics by league. The Bundesliga and Premier League have the highest average attendances, and La Liga and Serie A have higher average distances. The average home field advantage, measured by goal difference, is between 0.34 and 0.46 goals per game. Average home field advantages in expected goals are slightly lower, between 0.25 and 0.37 goals per game.

**Table 3.** Summary Statistics by League.

	Bundesliga	La Liga	Premier	Serie A
Attendance (thousands)	42.98	27.96	37.29	23.72
	(17.66)	(20.64)	(16.03)	(13.93)
Stadium Distance (km)	293.74	487.62	187.76	378.25
	(146.78)	(341.51)	(110.38)	(251.45)
Home Team Goals	1.64	1.59	1.57	1.52
	(1.36)	(1.36)	(1.32)	(1.25)
Home Team Expected Goals	1.59	1.43	1.43	1.48
	(0.82)	(0.75)	(0.79)	(0.80)
Away Team Goals	1.30	1.13	1.18	1.18
	(1.23)	(1.15)	(1.16)	(1.13)
Away Team Expected Goals	1.32	1.06	1.17	1.23
	(0.76)	(0.63)	(0.72)	(0.72)
Home-Away Goals Difference	0.34	0.46	0.38	0.34
	(1.94)	(1.85)	(1.84)	(1.69)
Home-Away Expected Goals Difference	0.28	0.37	0.26	0.25
	(1.23)	(0.98)	(1.21)	(1.19)

Notes: This table shows the differences between leagues across some key dimensions. Each cell represents the mean for that league, while the standard deviation is shown in parentheses. When calculating these statistics, an observation is a game and stadium distance for each game is the distance between the two home stadia. Expected goals are only calculated for the 2017–2018 season onwards, but all other variables start in the 2009–2010 season.

## Identification

Following the hiatus, no-fans policies were implemented in four major European soccer leagues. However, it is possible that non-fan factors could have impacted home field advantage. In this section, we discuss the potential threats to identification and how we address them.

The most direct and obvious potential confounding factor is that the COVID-19 pandemic may have affected home and away teams differently through infection. This is unlikely due to the low number of positive tests among soccer players. The players available to participate for the last quarter of the 2019–2020 season were largely the same as those that would have been available if not for the hiatus, excepting a small number of opt-outs and injuries. There is also the possibility that different regions had varying levels of exposure to COVID-19, and that these differences could affect the home field advantage. To address this concern, we control for the difference between total cases, as of March 31, in the home compared to away team regions, which we then interact with the no-fans policy. The inclusion of these controls does not qualitatively affect our results.

It is also possible that, by chance, weaker teams tend to be at home during this period. Although this is unlikely due to the random process involved in scheduling, we control for three different measures of home versus away team strength: difference in season-to-date points, difference in points in the last 4 games, and difference in points at the end of the previous season.^21^ Absent concerns with team-strength, there is still the concern that home field advantage fluctuates naturally throughout the season. The pressure on teams that are competing to stay above the relegation line or in the spots for European competitions could impact home field advantage. We address this by including (match) week by league fixed effects, which allow for differences in “seasonality” within each country.

**Figure 1.**
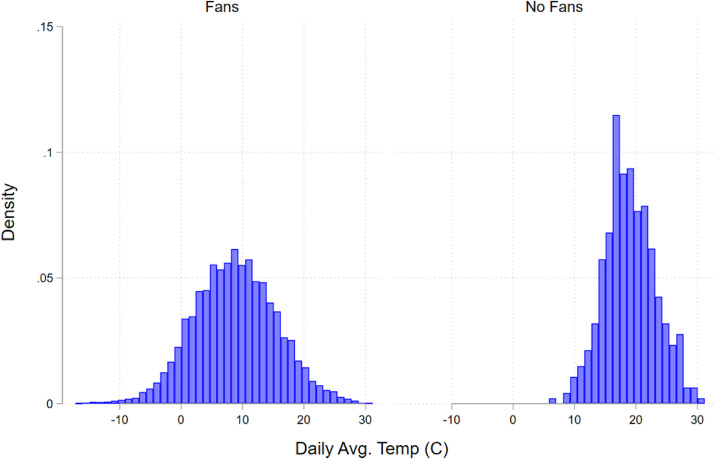
Weather distribution. Note: This figure shows the distribution of average daily temperatures for matches played when there are no fans compared to when there are fans. There is a shift in the distribution to higher temperatures, which reflects that matches were pushed into the summer.

There is also the possibility that the long hiatus of two or more months affected home and away teams differently. Within this channel are two subchannels, those being the long break itself and the differences in weather relative to when these games were originally scheduled. While there is some effect of these breaks on injuries (Ekstrand et al., 2019) and the outcome of matches (Jamil et al., 2020), there is no evidence that these breaks affect home and away teams differently. The primary impact of breaks on game performance is a decline in shot-to-goal conversion, but there is contradictory evidence that shot-to-goal conversion actually improved once play resumed from the COVID-induced hiatus (Cohen & Robinson, 2020). We abstract from potential differences in shot-to-goal conversion by analyzing expected goals in addition to realized goals, since expected goals reflect the quality and quantity of chances created regardless of the conversion rate. The second subchannel is that the delay in the season leads to different weather than in a normal season. The difference in average match-day temperature is evident in Figure 1, which plots the weather distribution for matches played with fans compared to without fans. There were two mitigation strategies undertaken by these four leagues to limit the weather burden on players; teams were allowed five substitutions per game instead of the usual three, and there were two water breaks per game in addition to the halftime intermission. It is unlikely that these slight changes would affect home field advantage directly, but they could mitigate any differences in home field advantage driven by warmer weather than when the games were originally scheduled to be played. To account for any effects of weather, however, we include flexible, non-linear controls for average temperature.

The last potential confounding factor is that one of the other two channels of home field ad- vantage — familiarity and travel — may have changed during this period. In stark contrast to many American sports leagues in 2020, games were played in the normal team stadia and were not moved to better mitigate the spread of COVID-19. Thus there was no difference in the familiarity effect as it is construed in the literature before and after the season was suspended. It is likely that travel was more onerous after the start of the pandemic; one might expect for away teams to perform worse relative to home teams due to more strenuous travel conditions.^22^ This potential difference in travel would lead us to understate any negative effects that the removal of fans have on home field advantage. Regardless, we control for distance traveled by the away team to best isolate the impact of fans on home field advantage.

## Empirical Framework

To recover a baseline estimate the effect of fans on home field advantage, we first estimate the following equation:

(1) $$y_{ijmsct}=\alpha +\beta Post_{msct}+\varepsilon_{ijmsc}$$

where the dependent variable, *y_ijmsct_*, represents the home minus away realized or expected goals difference in matchup *m* of season *s* in league *c* and week *t* between home team *i* and away team *j*. The intercept *α* is the home field advantage under normal circumstances, capturing the total effect of all three factors (fans, familiarity, and travel), and *Post_msct_* is an indicator variable equal to 1 if matchup *m* occurs after the implementation of a no-fans policy. The coefficient of interest *β* represents the change in home field advantage once these no-fans policies are implemented. Standard errors ε*_ijmsct_* are clustered at the matchup-by-season level to allow for correlation in the error term between observations involving the same two teams in the same season.^23^

The introduction of the no-fans policy induced by COVID-19 could coincide with changes in other factors that impact home field advantage. To mitigate any concerns of omitted variable bias, we estimate the following preferred specification:

(2) $$y_{ijmsct}=\alpha +\beta Post_{msct}+\gamma X_{ijmsct}^{1}+\eta X_{ijmsct}^{2}+\lambda_{ct}+\varepsilon_{ijmsct}$$

where we control for three sources of potential confounding factors. First, we address the concern that the introduction of the no-fans policy coincides with natural, within-season variation in home field advantage by including (match) week by league fixed effects, *λ_ct_*. Second, we address the role of travel and form on home field advantage in *X*^1^_ijmsct_, which includes distance, distance squared, point difference entering the match, point difference in last 4 games, and point difference in the previous season.^24^ Lastly, we account for two channels that are correlated with the no-fans policy — higher average temperatures and heterogeneous effects of COVID-19 by location — in *X*^2^_ijmsct_. The effect of temperature on home field advantage is likely to be non-linear and heterogeneous by league, due to differences in the typical climate faced. We allow for a flexible relationship between temperature and home field advantage by binning temperature at 5 degree Celsius intervals and allowing for a separate effect of temperature on home field advantage by league.^25^ Lastly, we control for the heterogeneous effects of COVID-19 by location by controlling for the difference in team region COVID-19 cases, as of March 31, interacted with *Post_msct_*.^26^

In addition to the above analysis of goal difference, we use an ordered logit model to investigate the effect of fans on match outcomes. For this specification, *y_msct_*, takes on 3 values based on if the home-team won (*y_msct_* = 2), drew (*y_msct_* = 1), or lost (*y_msct_* = 0) the match. We estimate the effect of the no-fans policy with the same controls shown in Equation 2 and graphically show the changes in predicted probability of match outcomes.

## Results

Column 1 of Table 4 shows that raw home field advantage decreased by 0.213 goals per game from a baseline of a 0.387 goals per game advantage for the home team.^27^ This represents a decrease of 55%. However, some fraction of this decrease is driven by factors other than the no-fans policy. Column 4 shows that the coefficient on treatment drops by approximately 18% but remains statistically significant at the 10% level. These changes in estimates and inference highlight the importance of other factors, such as weather, in establishing the effect of fans on home field advantage using the COVID-19 natural experiment.

**Table 4.** Impact of No Fans on Home Field Advantage - Realized Goals.

	(1)	(2)	(3)	(4)
	*G_H − A_*	*G_H − A_*	*G_H − A_*	*G_H − A_*
Baseline	0.387^∗∗∗^ [0.013]	0.387^∗∗∗^ [0.013]	0.336^∗∗∗^ [0.029]	0.342^∗∗∗^ [0.032]
No Fans	−0.213^∗∗^ [0.100]	−0.233^∗∗^ [0.103]	−0.188^∗∗^ [0.088]	−0.175^∗^ [0.105]
Points Difference			0.020^∗∗∗^ [0.001]	0.020^∗∗∗^ [0.001]
Diff in Recent Points (4 Games)			0.019^∗∗∗^ [0.004]	0.018^∗∗∗^ [0.005]
Diff in Prev. Season Points			0.022^∗∗∗^ [0.001]	0.022^∗∗∗^ [0.001]
Distance (thous. km)			0.163	0.137
			[0.112]	[0.121]
Distance (thous. km) Squared			−0.018	−0.007
			[0.081]	[0.084]
No Fans × Total Cases			−0.002	−0.002
			[0.009]	[0.008]
Controls	No	No	Yes	Yes
Week by League FE	No	Yes	No	Yes
Weather Controls	No	No	No	Yes
Observations	15,906	15,906	14,460	14,460
Games without Fans	408	408	408	408
Clusters	7,953	7,953	7,230	7,230

Significance: ***p* *< 0.01, **p < 0.05, *p < 0.1.

Notes: This table shows the change in home minus away goals when games are played with no fans (behind closed doors). This analysis uses data for all seasons from 2009–2020 in the following leagues: Bundesliga, Premier League, La Liga, and Serie A. The first row represents the estimated baseline home field advantage in terms of goals. The second row shows the estimated effect of the no-fans policy on home minus away goals. Each column shows a separate specification. The first column has no controls. The second column controls for league specific seasonality in home field advantage with (match) week by league fixed effects. The third column includes controls for team form and quality, distance traveled, and differences in COVID-19 cases by region interacted with the no-fans policy. The last column combines the previous two columns by including the controls of column 3 along with the fixed effects from column 2. In addition, we control for weather changes with 5 degree Celsius average temperature bin, *{<* 5*,* 0*–*5*,* 5–10*,* 10–15*,* 15–20*, >* 20*}*, by league fixed effects. Standard errors in brackets are clustered at the matchup by season level.

Column 1 of Table 5 shows that raw home field advantage, as measured by expected goals instead of realized goals, decreased by 64% from a 0.307 expected goal advantage for the home team to just 0.110 expected goals. Although the magnitude of the decrease is smaller than realized goals in absolute terms (0.197 xG as opposed to 0.213 G), it represents a larger fraction of the initial home field advantage (64% as opposed to 55%) because the initial home field advantage is smaller as measured by expected goals than realized goals. Column 4 of Table 5 shows that, although the effect of no fans on home field advantage, as measured by expected goals, decreases in magnitude when other controls are included, the effect remains statistically significant at the 1 percent level. This is in stark contrast to realized goals, in which the effect is only statistically significant at the 10 percent level when controls are included.^28^

**Table 5.** Impact of No Fans on Home Field Advantage - Expected Goals.

	(1)	(2)	(3)	(4)
	*xG_H − A_*	*xG_H − A_*	*xG_H − A_*	*xG_H − A_*
Baseline	0.307^∗∗∗^ [0.015]	0.308^∗∗∗^ [0.015]	0.271^∗∗∗^ [0.031]	0.285^∗∗∗^ [0.034]
No Fans	−0.197^∗∗∗^ [0.065]	−0.204^∗∗∗^ [0.074]	−0.184^∗∗∗^ [0.057]	−0.181^∗∗^ [0.086]
Points Difference			0.010^∗∗∗^ [0.002]	0.010^∗∗∗^ [0.002]
Diff in Recent Points (4 Games)			0.022^∗∗∗^ [0.005]	0.024^∗∗∗^ [0.005]
Diff in Prev. Season Points			0.017^∗∗∗^ [0.001]	0.016^∗∗∗^ [0.001]
Distance (thous. km)			0.121	0.069
			[0.122]	[0.134]
Distance (thous. km) Squared			0.007	0.022
			[0.089]	[0.092]
No Fans × Total Cases			0.000	0.000
			[0.005]	[0.005]
Controls	No	No	Yes	Yes
Week by League FE	No	Yes	No	Yes
Weather Controls	No	No	No	Yes
Observations	4,336	4,336	4,336	4,336
Games without Fans	408	408	408	408
Clusters	2,169	2,169	2,169	2,169

Significance: ***p < 0.01, **p < 0.05, *p < 0.1.

Notes: This table shows the change in home minus away expected goals when games are played with no fans (behind closed doors). This analysis uses data for all seasons from 2017–2020 in the following leagues: Bundesliga, Premier League, La Liga, and Serie A. The first row represents the estimated baseline home field advantage in terms of expected goals. The second row shows the estimated effect of the no-fans policy on home minus away expected goals. Each column shows a separate specification. The first column has no controls. The second column controls for league specific seasonality in home field advantage with (match) week by league fixed effects. The third column includes controls for team form and quality, distance traveled, and differences in COVID-19 cases by region interacted with the no-fans policy. The last column combines the previous two columns by including the controls of column 3 along with the fixed effects from column 2. In addition, we control for weather changes with 5 degree Celsius average temperature bin, *{<* 5*,* 0–5*,* 5–10*,* 10–15*,* 15–20*, >* 20*}*, by league fixed effects. Standard errors in brackets are clustered at the matchup by season level.

In addition to the primary specification, we also estimate the effect of no-fans policies on total goals per game. There is a small, statistically insignificant effect on total goals per game, shown in Table A1; even the larger estimated coefficient would only indicate a 3.5% increase in total goals per game. Therefore, the shift in goal difference is derived from approximately equal parts of fewer goals for the home team and more goals for the away team. The same is true for total expected goals, in which the estimated coefficients are positive but statistically insignificant and represent a tiny increase relative to the baseline.

**Figure 2.**
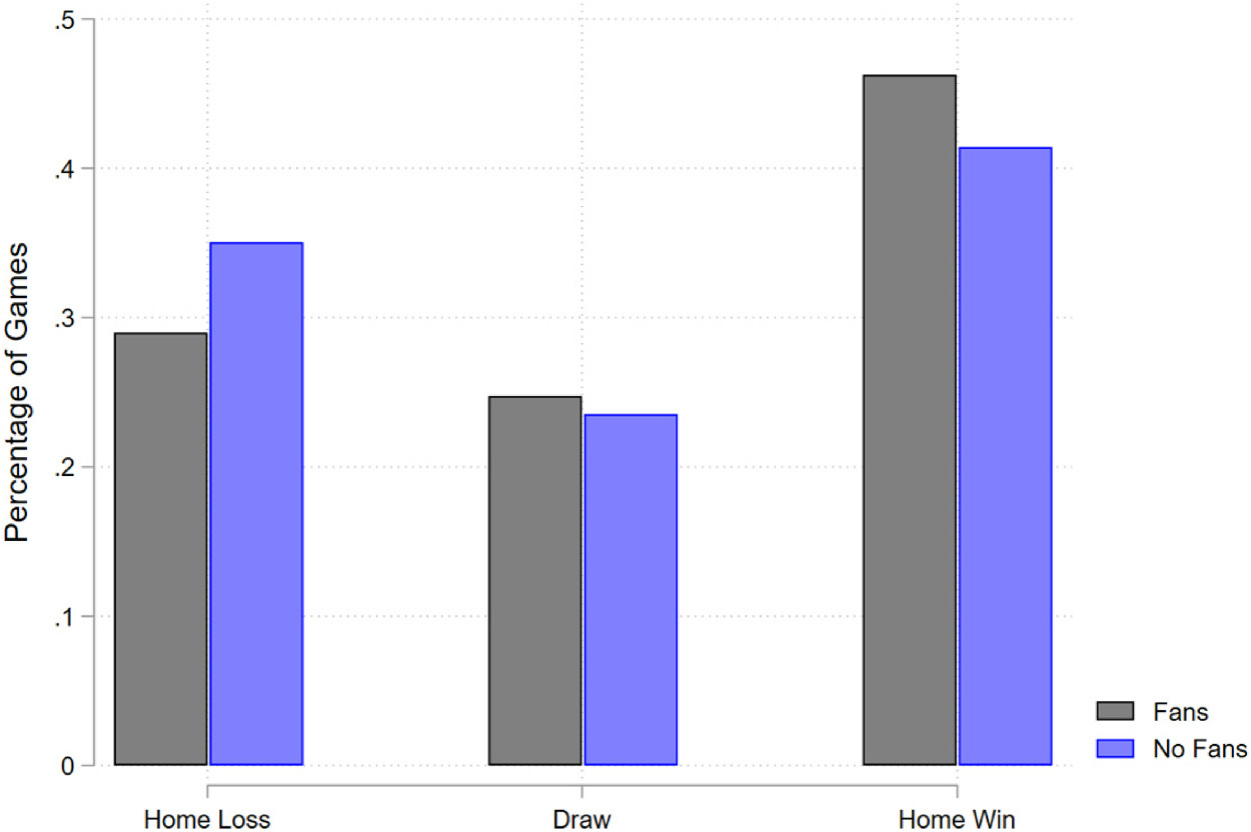
Change in Match Outcomes with No Fans. Note: This figure shows the distribution of match outcomes when there are no fans compared to when there are fans. There is little change in the percentage of games that end as a draw, but there are large differences for wins and losses.

**Figure 3.**
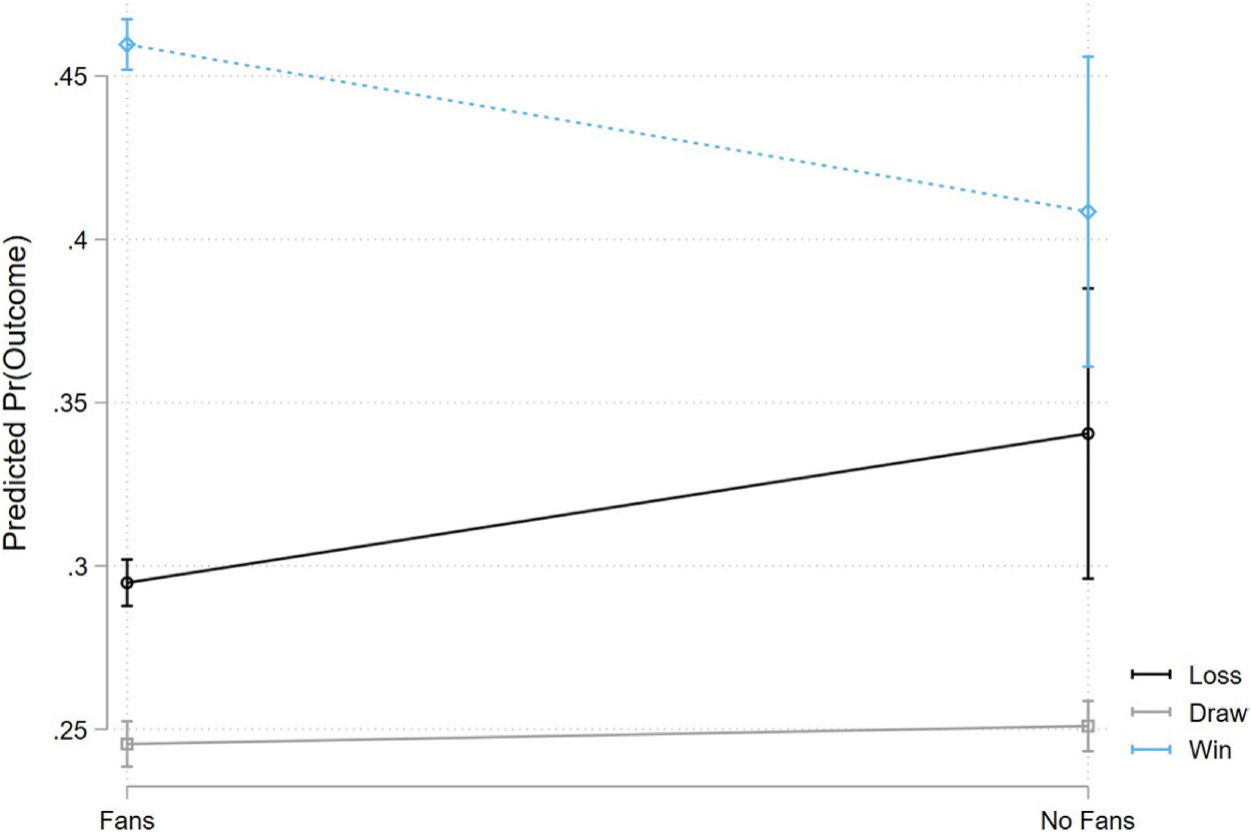
Effect of No Fans on Match Outcomes. Note: This figure shows the predicted probability of win, draw, and loss before and after the no-fans policy. Average predicted probabilities are calculated after fitting an ordered logistic model for match outcomes that matches the specification estimated in Column 4 of Tables 4 and 5. Specifically, we control for average home field advantage based on the (match) week of the season and temperature with week by league and 5 degree Celsius bin by league fixed effects. We also control for distance, distance squared, difference in cumulative cases interacted with the no-fans policy, and various measures of form and quality (i.e., differences in cumulative points). 95 percent confidence intervals are represented in the bands.

These shifts in goal difference due to the lack of fans manifest in fewer wins for the home teams. Figure 2 shows the simple shift in probability mass from home wins to home losses when fans are unable to attend games. Figure 3 shows the change in predicted probability of match outcomes from estimating an ordered logit. Importantly, it highlights that the lack of fans led to fewer home wins and more home losses, but the probability of a draw is unaffected, suggesting that fans are symmetrically pivotal: fans are approximately as likely to shift a result from a draw to a home win as they are from a home loss to a draw. Table A2 presents qualitatively similar results using a linear probability model instead of an ordered logit. We estimate a decrease in the probability of a home win and a precise null effect on the probability of a draw for the home team. Thus, there is a corresponding increase in probability for the omitted category (home loss). Approximately 5.4 percentage points are shifted from the probability of winning to the probability of losing.

In summary, we find that European soccer matches after the hiatus in the 2019–2020 season experienced a sharp decrease in the home field advantage across three key metrics. First, the actual difference between home and away goals decreased by 55 percent relative to the baseline. However, this decrease is only statistically significant at the 10 percent level when controlling for other factors that impact home field advantage that could be correlated with the no-fans policy. Second, this drop in actual goals is representative of changes in the home field advantage in terms of chance creation, as measured by expected goals, which decreased by approximately the same amount. Expected goals are not subject to as much noise as realized goals, so this decrease is a more reliable gauge of the true effect of fans on home field advantage.^29^ Lastly, these changes affect outcomes, as is shown by a decrease in the probability of a home win and an increase in the probability of a home loss.

## Conclusion

Using exogenous variation in attendance due to the COVID-19 pandemic, we find that home field advantage decreased by 64% as measured by expected goals when fans were not allowed in stadia. This result is robust to the inclusion of various fixed effects and controls for many potential confounding factors, including weather, team quality, form, and COVID-19 cases by region. Our estimates also suggest that the home field advantage dropped from 0.387 realized goals to 0.174 goals when no-fans policies were implemented to reduce the spread of the virus, but the estimated coefficient is only statistically significant at the 10 percent level because of the additional noise in the data generating process. These changes in home field advantage manifest in fewer home wins and more home losses when fans are not allowed in stadia. Using both an ordered logit regression and a linear probability model, we find that the lack of fans was important for match outcomes, suggesting that fans are themselves pivotal in how they affect sports.

One limitation of this paper is that we measure the effect of going from the “standard” number of fans down to zero. However, this standard level of fan attendance varies across leagues, teams, and even games, so the effect of marginal fans may vary in different contexts. Similarly, the impact of fans may be different for different types of competitions. Pollard and Pollard (2005) suggests that home advantage is smaller in national cup competitions, like the FA Cup in England, but larger in continental club competitions, like the UEFA Champions League. These different types of games also experience different levels of travel and fan attendance and may provide future evidence for the marginal effects of fans, as opposed to the total effect measured in this paper. Going forward, researchers may exploit plausibly exogenous variation in fan attendance for the 2020–2021 seasons, in which fan attendance was positive but limited to prevent the spread of the disease. The marginal effect of fans is a promising area of future research with real-world applications regarding optimal stadium capacity and ticket-pricing schema as soccer teams try to optimize on-field performance in addition to match-day revenue.

Another interesting note from the results is that the baseline home field advantage is approximately 24% smaller when measured by expected goals as opposed to realized goals. This may suggest that home-away differences in finishing (the act of converting scoring chances to goals) plays a significant role in the existing home field advantage. Future research may attempt to answer this question more directly, and analysis thereof may further explain and decompose home field advantage as it exists in soccer and other sports.

## Supplemental Material

sj-docx-1-jse-10.1177_15270025221100204 - Supplemental material for Do Fans Impact Sports Outcomes? A COVID-19 Natural Experiment^∗^ Click here for additional data file. Supplemental material, sj-docx-1-jse-10.1177_15270025221100204 for Do Fans Impact Sports Outcomes? A COVID-19 Natural Experiment^∗^ by Jeffrey Cross and Richard Uhrig in Journal of Sports Economics

## Notes

1. Home teams win approximately 47% of the time, draw 25% of the time, and lose 29% of the time

2. In addition, there may be some advantage regarding field preparation and style of play. In particular, FC Barcelona was known in the mid 2010's to keep their grass especially short and somewhat wet, such that short passes were easier to complete. Teams hosting FC Barcelona would sometimes prepare their grass to be long and dry in or- der to hamper the visitor's short passing style. https://www.dailymail.co.uk/sport/football/article-2600454/Barcelona- complain-UEFA-length-grass-Vicente-Calderon-ahead-Champions-League-showdown-Atletico.html. However, evidence for this type of advantage remains anecdotal to this point and is unidentified relative to the venue familiarity effect. Therefore, if one prefers to think of this more generally, then the venue familiarity effect can also include such factors as pitch preparation, the colors in the locker room, and the exact jerseys worn by each team.

3. See Table 1 for more details on how European soccer leagues responded to the COVID-19 pandemic.

4. Appendix B provides a more detailed description of how other sports and leagues responded to the COVID-19 pandemic and highlights the reasons why our setting is the best for analyzing the impact of fans on soccer games.

5. A same-stadium derby is a game between two teams that share a home stadium. The most famous example is AC Milan and Inter Milan, which both share the San Siro stadium. Such games occur twice per year, with each team technically “home” for one of the two games.

6. Many of these papers focus on changes in referee behavior as the potential mechanism (Bryson et al., 2021; Endrich & Gesche, 2020; Fischer & Haucap, 2021; Reade et al., 2021; Scoppa, 2021) with most finding that referees are less favorable to home teams without fans.

7. Nearly every winner and runner-up in the most prestigious European club competition, the Champions League, has come from these five leagues from the 1996–1997 through 2020–2021 seasons. The only exception is FC Porto, who won the Champions League in 2003–2004.

8. Each of these five major leagues paused their seasons within a few days of each other. The German Bundesliga was both the last league to stop play and the first to resume play. The last game before the hiatus was played on March 11th, and the first game after the restart was played on May 17th.

9. The exception here is the French Ligue 1, which decided to cancel the rest of the 2019–2020 season rather than resume play after the hiatus.

10. The similarities across leagues are not surprising since the heads of all five of the major European soccer leagues had weekly meetings starting in April.

11. Spain, for example, instituted a four phase program in the lead-up to the season, which was slightly different than what was instituted in other leagues.

12. The most publicized case of a player missing a game was Claudio Pizarro. He missed the game after the restart because his daughter tested positive. It is worth noting that there was an outbreak in a second division team in Germany, but no such flare-ups happened in any of the top division leagues considered here.

13. There are 20 teams in the English Premier League, Italian Serie A, and Spanish La Liga but only 18 teams in the German Bundesliga and French Ligue 1.

14. Due to scheduling conflicts arising from other competitions this does not always hold.

15. For more detailed description of how exactly expected goals are calculated, please refer to https://fbref.com/en/statsbomb/. Chances are considered similar based on distance, angle, goalkeeper and defender position, and shot type (shot with the foot or the head).

16. The realized goals could in fact be greater than four if the other team scores one or more own goals, which introduces even more noise.

17. FBref.com launched in June 2018 with league coverage for six nations: England, France, Spain, Italy, Germany, and the United States.

18. Specifically, we use the blended daily mean temperature dataset, which can be found here: https://www.ecad.eu/dailydata/predefined

19. In Figure A1, we show the distribution of distances between the stadium and station. Nearly 90% of observations have a station within 100 kilometers. Serie A matches tend to be farther from the weather stations, but the conclusions from our analysis are robust to the exclusion of Serie A.

20. Spatial granularity varies for each country in this analysis. Broken down by the Nomenclature of Territorial Units for Statistics (NUTS): Germany is level 1 (16 states), Spain is level 2 (19 autonomous communities), and Italy is level 3 (107 provinces). For England, the data is at the national level and so we are not able to estimate differential cases by team. Our results are robust to the exclusion of the English Premier League

21. Three points are earned for a won match, one point is earned for a drawn match, and no points are earned for a loss. For teams that are newly promoted to the top division, we set their “previous season points” as the points for the team that barely avoided relegation in the previous season.

22. If away teams are optimizing performance with respect to travel arrangements prior to the COVID-19 pandemic, then any additional restrictions placed upon those teams to avoid infection and outbreak could negatively affect the performance of away teams.

23. Note that for any matchup between two teams, there are two games played per season, one at each stadium. Thus, the home-away status in one game is perfectly negatively correlated with the home-away status of the same two teams in the reverse fixture.

24. Since we are controlling for previous season point difference, the first season in our data, 2009–2010, is dropped. For teams that are promoted, the previous season points value entered is the lowest points value for a team that stayed up in the previous season. Our results are quantitatively robust to the exclusion of this variable and estimation on the full sample.

25. This approach of binning is similar to what has been used when estimating the effect of temperature on mortal-ity Deschênes and Greenstone (2011). In this context, we employ 6 temperature bins *{<* 0*,* 0–5*,* 5–10*,* 10–15*,* 15–20*, >* 20*}* that are interacted with league indicator variables to account for potential non-linearity and heterogeneous effects by league.

26. Our results are quantitatively robust to the use of either new cases on March 31 or total cases divided by population instead of overall cases.

27. Results, shown in Table A3 are qualitatively similar using only the restricted sample for which expected goals data are available.

28. Realized goals are the result of a noisier process than expected goals. Each shot can only take on the values of zero or one realized goals, leading to larger error terms, whereas shots may relate to a value of expected goals anywhere between zero and one. This relates to the fact that expected goals are a better predictor of future performance than actual goals, as is discussed in Section 2.3.

29. The effect of no-fans policies on expected goal difference is significant at the 1 percent level when controls are concluded, compared to the 10 percent level for realized goal difference.

## Appendices

### A Additional Tables and Figures

**Table A1.** Impact of No Fans on Total Goals per Game.

	(1)	(2)	(3)	(4)
	*G_total_*	*G_total_*	*xG_total_*	*xG_total_*
Baseline	2.766^∗∗∗^ [0.014]	2.732^∗∗∗^ [0.034]	2.661^∗∗∗^ [0.016]	2.624^∗∗∗^ [0.036]
No Fans	0.099	−0.032	0.049	0.022
	[0.079]	[0.093]	[0.051]	[0.074]
Points Difference		0.001		−0.000
		[0.002]		[0.002]
Diff in Recent Points (4 Games)		0.001		0.008^∗^
		[0.005]		[0.005]
Diff in Prev. Season Points		0.003^∗∗∗^ [0.001]		0.001^∗∗^ [0.001]
Distance (thous. km)		0.119		0.106
		[0.129]		[0.131]
Distance (thous. km) Squared		0.009		0.034
		[0.087]		[0.082]
No Fans × Total Cases		0.000		0.002
		[0.008]		[0.005]
Controls	No	Yes	No	Yes
Week by League FE	No	Yes	No	Yes
Observations	15,906	14,460	4,336	4,336
Games without Fans	408	408	408	408
Clusters	7,953	7,230	2,169	2,169

Significance: ***p < 0.01, **p < 0.05, *p < 0.1.

Notes: This table shows the change in goals and total goals when games are played with no fans (behind closed doors). This analysis uses data for all seasons from 2009–2020 for the first two columns and 2017–2020 for the last two columns in the following leagues: Bundesliga, Premier League, La Liga, and Serie A. The first row represents the estimated baseline average total goals or total expected goals. The second row shows the estimated effect of the no-fans policy on total goals or total expected goals. Each column shows a separate specification or dependent variable. The first and third columns have no controls. The second and fourth columns control for league specific seasonality in home field advantage with (match) week by league fixed effects. They also include controls for team form and quality, distance traveled, and differences in COVID-19 cases by region interacted with the no-fans policy. In addition, we control for weather changes with 5 degree Celsius average temperature bin, *{<* 5*,* 0–5*,* 5–10*,* 10–15*,* 15–20*, >* 20*}*, by league fixed effects. Standard errors in brackets are clustered at the matchup by season level.

**Table A2.** Impact of No Fans on Home Field Advantage - Outcomes.

	(1) Win	(2) Win	(3) Draw	(4) Draw
Baseline	0.463^∗∗∗^ [0.004]	0.446^∗∗∗^ [0.009]	0.247^∗∗∗^ [0.003]	0.246^∗∗∗^ [0.008]
No Fans	−0.048^∗^	−0.057^∗∗^	−0.012	0.023
	[0.025]	[0.027]	[0.021]	[0.025]
Points Difference		0.004^∗∗∗^		0.000
		[0.000]		[0.000]
Diff in Recent Points (4 Games)		0.005^∗∗∗^ [0.001]		−0.003^∗∗^ [0.001]
Diff in Prev. Season Points		0.005^∗∗∗^ [0.000]		−0.001^∗∗∗^ [0.000]
Distance (thous. km)		0.058^∗^		−0.005
		[0.035]		[0.032]
Distance (thous. km) Squared		−0.016		0.001
		[0.023]		[0.023]
No Fans × Total Cases		0.003		−0.005^∗∗^
		[0.002]		[0.002]
Controls	No	Yes	No	Yes
Week by League FE	No	Yes	No	Yes
Observations	15,906	14,460	15,906	14,460
Games without Fans	408	408	408	408
Clusters	7,953	7,230	7,953	7,230

Significance: ***p < 0.01, **p < 0.05, *p < 0.1.

Notes: This table shows the change in the probability of a home team win or draw when games are played with no fans (behind closed doors). This analysis uses data for all seasons from 2009–2020 in the following leagues: Bundesliga, Premier League, La Liga, and Serie A. The first row represents the estimated baseline probability of a home team win or draw. The second row shows the estimated effect of the no-fans policy on the match outcomes. Each column shows a separate specification or dependent variable. The first and third columns have no controls. The second and fourth columns control for league specific seasonality in home field advantage with (match) week by league fixed effects. They also include controls for team form and quality, distance traveled, and differences in COVID-19 cases by region interacted with the no-fans policy. In addition, we control for weather changes with 5 degree Celsius average temperature bin, *{<* 5*,* 0–5*,* 5–10*,* 10–15*,* 15–20*, >* 20*}*, by league fixed effects. Standard errors in brackets are clustered at the matchup by season level.

**Table A3.** Restricted Sample Realized Goals.

	(1)	(2)	(3)	(4)
	*G_H − A_*	*G_H − A_*	*G_H − A_*	*G_H − A_*
Baseline	0.329^∗∗∗^ [0.026]	0.336^∗∗∗^ [0.026]	0.292^∗∗∗^ [0.052]	0.282^∗∗∗^ [0.058]
No Fans	−0.174^∗^ [0.104]	−0.245^∗∗^ [0.118]	−0.158^∗^ [0.091]	−0.273^∗^ [0.142]
Points Difference			0.014^∗∗∗^ [0.003]	0.014^∗∗∗^ [0.003]
Diff in Recent Points (4 Games)			0.031^∗∗∗^ [0.008]	0.033^∗∗∗^ [0.008]
Diff in Prev. Season Points			0.023^∗∗∗^ [0.001]	0.022^∗∗∗^ [0.001]
Distance (thous. km)			0.170	0.251
			[0.198]	[0.218]
Distance (thous. km) Squared			−0.068	−0.099
			[0.138]	[0.149]
No Fans × Total Cases			0.003	0.003
			[0.008]	[0.009]
General Controls	No	No	Yes	Yes
Week by League FE	No	Yes	No	Yes
Weather Controls	No	No	No	Yes
Observations	4,336	4,336	4,336	4,336
Games without Fans	408	408	408	408
Clusters	2,169	2,169	2,169	2,169

Significance: ***p < 0.01, **p < 0.05, *p < 0.1.

Notes: This table shows the change in home minus away goals when games are played with no fans (behind closed doors). This analysis uses data for all seasons from 2017–2020 in the following leagues: Bundesliga, Premier League, La Liga, and Serie A. The first row represents the estimated baseline home field advantage in terms of goals. The second row shows the estimated effect of the no-fans policy on home minus away goals. Each column shows a separate specification. The first column has no controls. The second column controls for league specific seasonality in home field advantage with (match) week by league fixed effects. The third column includes controls for team form and quality, distance traveled, and differences in COVID-19 cases by region interacted with the no-fans policy. The last column combines the previous two columns by including the controls of column 3 along with the fixed effects from column 2. In addition, we control for weather changes with 5 degree Celsius average temperature bin, *{<* 5*,* 0– 5*,* 5–10*,* 10–15*,* 15–20*, >* 20*}*, by league fixed effects. Standard errors in brackets are clustered at the matchup by season level.

### B Comparison to North American Sports Leagues

The impact of COVID-19 on the four European soccer leagues studied here differ in some important respects compared to the major North American sports leagues. After a hiatus similar to that in European soccer, North American leagues that were already in the midst of their respective seasons all resumed play in a bubble format designed to minimize exposure to the disease.^30^ Games were played without fans and at neutral sites, as opposed to the originally scheduled stadia. In some cases, planned games were canceled due to the lost time. These bubbles were largely successful in preventing COVID-19 spread, although some whole teams (primarily in Major League Soccer) did experience outbreaks that led to cancellations. Even in the National Basketball Association bubble, many more players missed many more games due to COVID-19 than all four European soccer leagues combined, either from the actual sickness or refusal to play under the bubble conditions.

North American sports leagues that had not yet begun their seasons did not implement a bubble format but instead adopted a structure somewhat similar to European soccer. National Football League and Major League Baseball games were played in originally scheduled stadia, but the National Football League did actually allow some fans to attend. Major League Baseball drastically shortened the season^31^ and implemented non-trivial rule changes to actually gameplay, adjusting extra innings, doubleheaders, and designated hitter usage. Additionally, there were many outbreaks in some teams that led to canceled and postponed games, in stark contrast to the European soccer leagues.
