# Supplementary material for: Do Fans Impact Sports Outcomes? A COVID-19 Natural Experiment
Source: J Sports Econom. 2023 Jan;24(1):3–27. doi: 10.1177/15270025221100204 (PMC9121146; doi:10.1177/15270025221100204)
Supplement: sj-docx-1-jse-10.1177_15270025221100204 - Supplemental material for Do Fans Impact Sports Outcomes? A COVID-19 Natural Experiment∗ [file sj-docx-1-jse-10.1177_15270025221100204.docx]

Data from matches can be accessed from the soccer statistics website FBref.com. As a robustness check, we also control for weather using the European Climate Assessment and Dataset and the prevalence of COVID-19 using a spatially-resolved dataset from Carleton et al (2021).
